# Regression models for censored time-to-event data using infinitesimal jack-knife pseudo-observations, with applications to left-truncation

**DOI:** 10.1007/s10985-023-09597-5

**Published:** 2023-05-08

**Authors:** Erik T. Parner, Per K. Andersen, Morten Overgaard

**Affiliations:** 1 Section for Biostatistics, Aarhus University, Bartholins Allé 2, 8000 Aarhus C, Denmark; 2 Section of Biostatistics, University of Copenhagen, Øster Farimagsgade 5, 1014 Copenhagen K, Denmark

**Keywords:** Competing risks, Cumulative incidence, Cumulative risk, Left-truncation, Pseudo-observations

## Abstract

Jack-knife pseudo-observations have in recent decades gained popularity in regression analysis for various aspects of time-to-event data. A limitation of the jack-knife pseudo-observations is that their computation is time consuming, as the base estimate needs to be recalculated when leaving out each observation. We show that jack-knife pseudo-observations can be closely approximated using the idea of the infinitesimal jack-knife residuals. The infinitesimal jack-knife pseudo-observations are much faster to compute than jack-knife pseudo-observations. A key assumption of the unbiasedness of the jack-knife pseudo-observation approach is on the influence function of the base estimate. We reiterate why the condition on the influence function is needed for unbiased inference and show that the condition is not satisfied for the Kaplan–Meier base estimate in a left-truncated cohort. We present a modification of the infinitesimal jack-knife pseudo-observations that provide unbiased estimates in a left-truncated cohort. The computational speed and medium and large sample properties of the jack-knife pseudo-observations and infinitesimal jack-knife pseudo-observation are compared and we present an application of the modified infinitesimal jack-knife pseudo-observations in a left-truncated cohort of Danish patients with diabetes.

**Supplementary Information:**

The online version contains supplementary material available at 10.1007/s10985-023-09597-5.

## Introduction

The Cox regression model for hazards is a common analysis of time-to-event data. In recent years, regression model methods have been developed for analysis of other parameters than hazard ratios. Such parameters include contrasts between cumulative incidences, restricted means, and number of life years lost due to specific causes of death (Klein et al. 2007; Andersen et al. 2004; Scheike et al. 2008; Andersen 2013). A flexible framework has been established by creating a transformation of the time-to-event data, often called pseudo-observations, which are analyzed using a simple estimating equation for a generalized linear model. A common variant of the pseudo-observations is based on jack-knife residuals (Andersen et al. 2003).

The framework for the pseudo-observation method is regression analysis for $\textrm{E}(V|Z)$, where *V* is a function of the time-to-event data of interest and *Z* is a vector of covariate. The jack-knife pseudo-observations are based on a well-behaved estimate of $\theta =\textrm{E}(V)$. For survival data with event time *T*, say, $V=1(T>t)$ is chosen if the purpose of the regression is to analyze the survival probability at a pre-defined time *t*, $\textrm{E}(V|Z)=\text {P}(T>t|Z)$. Here the Kaplan–Meier estimate is used as the base estimate. For competing risk data $(T,\Delta )$, where $\Delta =1$ denotes the event of interest and $\Delta =2,\ldots ,d$ denote the competing events, $V=1(T\le t,\Delta =1)$ is chosen for regression models of the cumulative risk for the type-1 event, $\textrm{E}(V|Z)=\text {P}(T\le t, \Delta =1|Z)$. Here the Aalen–Johansen estimate is used as the base estimate. Let *X* denote the observed censored time-to-event data, i.e., for a right-censoring time *C* the observed time-to-event for the competing risk set-up $X=(\tilde{T}, \tilde{\Delta })$, where the censored event time is $\tilde{T}=T\wedge C$ and event type is $\tilde{\Delta }=\Delta 1\{T\le C\}$. Let $(X_1,Z_1),\ldots , (X_n,Z_n)$ denote an i.i.d. sample of observations with time-to-event data $X_i$ and a vector of covariate $Z_i$. Further, let $\hat{\theta }_n$ denote the base estimate of $\theta$ based on the full sample $X_1,\ldots , X_n$ and let $\hat{\theta }_n^{(i)}$ denote the similar estimate based on the sample $X_1,\ldots ,X_{i-1}, X_{i+1},\ldots ,X_{n}$, i.e., leaving out $X_i$, then the jack-knife pseudo-observation for $V_i$ is defined as

1 $$\begin{aligned} \hat{\theta }_{n,i} = n\hat{\theta }_n-(n-1)\hat{\theta }_n^{(i)}. \end{aligned}$$

Suppose a regression model $\textrm{E}(V|Z)=\mu (\beta _0;Z)$ is specified, where $\mu (\beta ; Z) = \mu (\beta ^T Z)$ typically is the inverse of the link function in a generalized linear regression. Estimates of $\beta _0$ are then obtained based on $\hat{\theta }_{n,1},\ldots , \hat{\theta }_{n,n}$ by solving the estimating equation

2 $$\begin{aligned} \sum _{i=1}^n A(\beta ; Z_i)\{\hat{\theta }_{n,i}-\mu (\beta ; Z_i )\} = 0, \end{aligned}$$

where $A(\beta ;Z_i)$ is a vector function depending only on the regression parameters and covariates. The asymptotic distribution of $\hat{\beta }_n$ has been studied for base estimates which are functionals of sample averages of the observations, $F_n=\frac{1}{n}\sum _{i=1}^n\delta _{X_i}$, i.e. estimates of the form $\hat{\theta }_n=\phi (F_n)$ (Jacobsen and Martinussen 2016; Overgaard et al. 2018). Here $\delta _{X_i}$ is a functional of the time-to-event data, usually a counting process representation of the time-to-event data, see Section 2.1 for further details. The robust Huber–White variance estimate is often a good approximation of the variance of the regression parameter estimate $\hat{\beta }_n$ solving (2). However, the Huber–White variance estimate ignores the correlation between the pseudo-observations. The correct asymptotic variance is more complicated and involves both first and second derivative of the base estimate function $\phi$. In addition to the smoothness of the base estimate function, the jack-knife pseudo-observation method is based on a central assumption of the base estimate: the first order influence function at a time-to-event observation *x*,

$$\begin{aligned} \dot{\phi }_F(x) = \phi _F^\prime (\delta _x-F) \end{aligned}$$

should satisfy $\text {E}(\dot{\phi }_F(X_i)|Z_i)=\text {E}(V_i|Z_i)-\text {E}(V_i)$ (Overgaard et al. 2018), see Section 2.1 for further details. Here, *F* denotes the limit of $F_n$ when it exists, and often $\phi (F)=\theta =\text {E}(V_i)$. Further details on differentiability of functionals and properties of influence functions are given in Section A of the supplement. Overgaard et al. (2019) showed that the influence function condition is satisfied concerning base estimating functions that can be written in an inverse probability of censoring weighting form; otherwise, the assumption needs to be checked case by case. It is worth reiterating why the condition on the influence function is needed for unbiased inference.

A major advantage of the jack-knife pseudo-observation method is its general formulation for performing regression analyses, and the method can often be implemented using standard statistical software. One limitation is that it is time-consuming, as the base estimate needs to be recalculated for each observation. Considerable efforts have been made to provide efficient implementation for computing the jack-knife pseudo-observations such as the implementations for cumulative incidences, restricted means, and numbers of life years lost due to specific causes of death in the software programs R, SAS and Stata in Klein et al. (2008); Parner and Andersen (2010); Overgaard et al. (2015); Gerds (2019); Therneau (2021).

Jaeckel (1972) suggested using infinitesimal jack-knife residuals for variance estimation, which for each subject is an estimate of the influence function $\dot{\phi }_F(X_i)$. Efron (1982) considered a bootstrap variance using the infinitesimal jack-knife residuals. We consider using pseudo-observations based on the infinitesimal jack-knife residuals for regression analysis. One advantage of the infinitesimal jack-knife pseudo-observations is that they are much faster to compute, making the non-parametric bootstrap method attractive for variance estimation. We showed that the jack-knife and infinitesimal jack-knife pseudo-observations are asymptotically equivalent when the base estimator is a sufficiently smooth function of the time-to-event data. We compared the computational speed of the jack-knife and infinitesimal jack-knife pseudo-observations and present medium and large sample comparison of the two methods in simulations. The infinitesimal jack-knife pseudo-observations are easy to modify to model assumptions. We showed that for a cohort with left-truncation, the influence function of the base Kaplan–Meier estimator does not have the desired expected value, although the use of jack-knife pseudo-observations for a left-truncated cohort has been reported in the literature (Grand et al. 2019; Shen 2021) and currently implemented in statistical packages (Overgaard et al. 2015; Therneau 2021). We suggest a modification of the infinitesimal jack-knife pseudo-observations that together with inverse probability of sampling weights applied to the estimation equation provides unbiased estimates for a cohort with left-truncation. Finally, we compared the jack-knife and modified infinitesimal jack-knife pseudo-observations using data on Danish patients with diabetes.

## Method

The motivation for the infinitesimal jack-knife pseudo-observations, as well as the intuition of the condition on the influence function for unbiasedness, comes from a property of the jack-knife pseudo-observations proved in Overgaard et al. (2017). Overgaard and colleagues showed that under regularity conditions the jack-knife pseudo-observations satisfy

3 $$\begin{aligned} \hat{\theta }_{n,i}=\phi (F) +\dot{\phi }_F(X_i)+\frac{1}{n-1}\sum _{j\ne i}\ddot{\phi }_F(X_i,X_j)+o_{\text {P}}(n^{-1/2}), \end{aligned}$$

uniformly in $i=1,\ldots ,n$, where $\ddot{\phi }_F(\cdot )$ is the second order influence function at two time-to-event observations $x_1$ and $x_2$,

$$\begin{aligned} \ddot{\phi }_F(x_1,x_2) = \phi _F^{\prime \prime }(\delta _{x_1}-F,\delta _{x_2}-F). \end{aligned}$$

Here $o_{\text {P}}$ refers to convergence in probability, i.e., for a sequence of variables $\{Y_n\}_{n\ge 1}$ we write $Y_n=o_{\text {P}}(a_n)$ when $a_n^{-1}Y_n$ converge in probability towards zero. It is important that the approximation occurs uniformly in $i=1,\ldots ,n$, as the approximation is used for each observation *i*. One can view (3) as the approximate transformation of the original time-to-event dataset created by the pseudo-observations. The condition on the influence function of the base estimate appears when the approximation (3) is inserted in the estimation equation (2) to ensure that the estimation function has a mean zero at $\beta =\beta _0$. First, note that the second order influence function satisfies $\text {E}(\ddot{\phi }_F(X,x))=0$ for all observations *x*, implying that $\text {E}(A(\beta ;Z_i) \ddot{\phi }(X_i,X_j))=0$. The remaining part of the mean estimation function is then

$$\begin{aligned}&\text {E}(A(\beta _0; Z_i)\{\phi (F)+\dot{\phi }_F(X_i)-\mu (\beta _0; Z_i )\} ) \\&=\text {E}(A(\beta _0; Z_i)\{\phi (F)+\text {E}(\dot{\phi }_F(X_i)|Z_i)-\mu (\beta _0; Z_i )\} ), \end{aligned}$$

which is zero if $\text {E}(\dot{\phi }_F(X_i)|Z_i)=\mu (\beta _0; Z_i )-\phi (F)=\text {E}(V_i|Z_i)-\text {E}(V_i)$. Thus, the condition on the influence function ensures that the estimation equation (2) has a mean zero when $\beta =\beta _0$.

The idea of the infinitesimal jack-knife pseudo-observations is to use an estimate of $\phi (F) +\dot{\phi }_F(X_i)$ as pseudo-observations used for regression analysis. Based on the infinitesimal jack-knife residuals of Jaeckel (1972), we define pseudo-observations

4 $$\begin{aligned} \hat{\theta }_{n,i}^{\text {IJ}}&=\phi (F_n)+\dot{\phi }_{F_n}(X_i). \end{aligned}$$

In the supplement Section B, we show that when the base estimator is a sufficiently smooth functional of the time-to-event data, $\hat{\theta }_{n,i}-\hat{\theta }_{n,i}^{\text {IJ}}=o_\text {P}(n^{-1/2})$, uniformly in $i=1,...,n$. The smoothness condition was verified for the Kaplan–Meier and the Aalen–Johansen estimator in Overgaard et al. (2017). Thus, $\hat{\theta }_{n,i}^{\text {IJ}}$ may replace $\hat{\theta }_{n,i}$ in the estimating equation with the same asymptotic properties. Using either form of pseudo-observations, and assuming standard asymptotic regularity conditions as stated in Overgaard et al. (2017), the solution $\hat{\beta }_n$ to the estimation equation (2) satisfies: $\sqrt{n}(\hat{\beta }_n-\beta _0)$ converges in distribution to a mean zero normal distribution with variance $M^{-1}\Sigma (M^{-1})^T$ with

$$\begin{aligned} M=\textrm{E}\left\{ A(\beta _0;Z)\frac{\partial }{\partial \beta }\mu (\beta ;Z)\Big |_{\beta =\beta _0}\right\} \end{aligned}$$

and

5 $$\begin{aligned} \Sigma = \textrm{Var}\{h_0(X,Z)+h_1(X)\}, \end{aligned}$$

where

$$\begin{aligned} h_0(X,Z)&=A(\beta _0;Z)\{\phi (F)+\dot{\phi }_F(X)-\mu (\beta _0;Z)\} \\ h_1(x)&=\textrm{E}\{A(\beta _0;Z)\ddot{\phi }_F(X,x)\}. \end{aligned}$$

Note that the variance depends on the first order influence function in the $h_0$ term and the second order influence function in the $h_1$ term. The variance was derived for the Aalen–Johansen functional in Overgaard et al. (2018). The Huber–White robust variance estimate is an estimate of $\textrm{Var}\{h_0(X,Z)\}$, ignoring the $h_1$ part of $\Sigma$, and is therefore biased. The bias has been shown to be upwards under the model for pseudo-observations based on the Kaplan–Meier and Aalen–Johansen estimators. As such, the Huber–White robust variance estimate will be conservative in these cases. However, the Huber–White robust variance estimate has both theoretically and in simulation studies been demonstrated to be a good approximation of the asymptotic variance, unless the covariates have a strong effect on the outcome and there is a large proportion of censored observations (Jacobsen and Martinussen 2016; Overgaard et al. 2018).

For the infinitesimal jack-knife pseudo-observations, the influence function estimate $\dot{\phi }_{F_n}(x)$ is computed only once for all *x* and evaluate at $x=X_i$ to compute the infinitesimal jack-knife pseudo-observation for subject *i*, making the infinitesimal jack-knife pseudo-observations much faster to compute than the jack-knife pseudo-observations. It is therefore attractive to use non-parametric bootstrap to estimate the asymptotic variance. Another advantage of the infinitesimal jack-knife pseudo-observations is that the method can be modified to assumptions on the censoring and selection, as we will demonstrate in the next section.

### Competing risk data

Let *T* denote the time of event, $\Delta \in \{ 1, \ldots , d\}$ the type of event and *Z* a vector of covariates. Consider regression models of the cumulative 1-event risk at time *t*, $V=1\{T\le t,\Delta =1\}$, $\textrm{E}(V)=\text {P}(T\le t,\Delta =1)=:F_1(t)$, and $\textrm{E}(V|Z)=:F_1(t|Z)=\mu (\beta _0; Z)$. Let *C* be a right-censoring time and denote the censored event time $\tilde{T}=T\wedge C$ and event type $\tilde{\Delta }=\Delta 1\{T\le C\}$. The observed time-to-event data is then $X=(\tilde{T}, \tilde{\Delta })$. If the data is subject to left-truncation, we only observe subjects where the entry time *L* is smaller than $\tilde{T}$, $L\le \tilde{T}$. We assume that *L*, *C*, and $(T,\Delta ,Z)$ are mutually independent. As a model of *n* observations, let $(\tilde{T}_i,\tilde{\Delta }_i,Z_i,L_i)$ be independent replicates of $(\tilde{T},\tilde{\Delta },Z,L)$ and consider a sample of *n* subjects with $L_i\le \tilde{T}_i$.

#### A cohort without left-truncation

The infinitesimal jack-knife pseudo-observations are based on specifying the counting process representation, $\delta _{X_i}$, of the time-to-event data, identifying the base estimate $\phi$ as a functional of $F_n=\frac{1}{n}\sum _{i=1}^n\delta _{X_i}$ and finally computing the influence function of $\phi$. Define event at-risk indicator process $Y_i(s)=1(s\le \tilde{T}_i)$, censoring at-risk indicator process $Y_{\text {c},i}(s)=1(s<\tilde{T}_i)+1(s=\tilde{T}_i,\tilde{\Delta }_i=0)$ that handles tied event and censoring times, as well as counting processes $N_{i,j}(s)=1(\tilde{T}_i\le s,\tilde{\Delta }_i=j)$ for censoring $j=0$ and event type $j=1,\ldots ,d$. The Aalen–Johansen estimate is a function of averages of $\delta _{X_i}=(Y_i(\cdot ),Y_{\text {c},i}(\cdot ),N_{i,j}(\cdot ),j=0,\ldots ,d)^T$ with expectation $F=(H,H_\text {c},H_j,j=0,\ldots ,d)^T$, where $H(s)=\text {E}(Y(s))=\text {P}(s\le \tilde{T}_i)$, $H_\text {c}(s)=\text {E}(Y_{\text {c}}(s))=\text {P}(s<\tilde{T}_i)+\text {P}(s=\tilde{T}_i,\tilde{\Delta }_i=0)$ and $H_j(s)=\text {E}(N_{i,j}(s))=\text {P}(\tilde{T}_i\le s,\tilde{\Delta }_i =j)$, $j=0,\ldots ,d$. Similar to Overgaard et al. (2017), we assume that $H_\text {c}(t)>0$, where *t* is the analysis time point of interest. Let $\hat{H}_n(s)=\frac{1}{n}\sum _{i=1}^n Y_i(s)$, $\hat{H}_{n,\text {c}}(s)=\frac{1}{n}\sum _{i=1}^n Y_{\text {c},i}(s)$ and $\hat{H}_{n,j}(s)=\frac{1}{n}\sum _{i=1}^n N_{i,j}(s)$ for $j=0,\ldots ,d$ denote their empirical versions. The cumulative censoring hazard $\Lambda _0(s)=\int _0^s \frac{1}{H_\text {c}(u)}\text {d}H_0(u)$ is estimated by the Nelson–Aalen estimate $\hat{\Lambda }_{n,0}(s)=\int _0^s \frac{1}{\hat{H}_{n,\text {c}}(u)}\text {d}\hat{H}_{n,0}(u)$ and an estimate of the survival function for the censoring distribution  is the Kaplan–Meier estimate , where  is the product integral. Similarly, the cumulative *j*-event hazard $\Lambda _j(s)=\int _0^s \frac{1}{H(u)}\text {d}H_j(u)$ is estimated by $\hat{\Lambda }_{n,j}(s)=\int _0^s \frac{1}{\hat{H}_n(u)}\text {d}\hat{H}_{n,j}(u)$ and an estimate of the survival function  is . The Aalen–Johansen estimate of the cumulative event risk, $F_j(s)=\int _0^s \frac{1}{G(u-)} \text {d}H_j(u)$, in its inverse probability of censoring weighted (IPCW) form is $\hat{F}_{n,j}(s)=\int _0^s \frac{1}{\hat{G}_n(u-)}\text {d}\hat{H}_{n,j}(u)$. The Aalen–Johansen estimate $\hat{F}_{n,1}$ is thus a function, $\phi$, of the sample average of the data, $F_n=\frac{1}{n}\sum _{i=1}^n\delta _{X_i}$. Evaluating $\phi$ at *F* we obtain $F_1(t)$.

The first order influence function is defined as $\dot{\phi }_F( x)=\phi _{F}^\prime (\delta _x-F)$, where the first derivative at *f* in direction *g* is $\phi _f^\prime (g)=\frac{\partial }{\partial s}\phi (f +sg)\big |_{s=0}$. As stated in Overgaard et al. (2017), the influence function for the Aalen–Johansen function is

6 $$\begin{aligned} \dot{\phi }_F(X)=\int _0^t \frac{1}{G(s-)}\text {d}\{ N_{1}(s)-H_1(s)\} +\int _0^t \frac{F_1(t)-F_1(s)}{H(s+)}\text {d}M_{0}(s), \end{aligned}$$

where $M_{0i}(s)=N_{0i}(s)-\int _0^sY_{\text {c},i}(u)\text {d}\Lambda _0(u)$. The at-risk probability function $H(s+)$ can also be written as *S*(*s*)*G*(*s*), where *S* is the event survival function and *G* is the survival function for the censoring distribution. From the martingale property of $M_0$, in the filtration where the covariate information is also included, it is easily verified that $\text {E}(\dot{\phi }_F(X)|Z)=F_{1}(t|Z)-F_1(t)$. It follows that

7 $$\begin{aligned} \phi (F)+\dot{\phi }_F(X) =\int _0^t \frac{1}{G(s-)}\text {d}N_{1}(s) +\int _0^t \frac{F_1(t)-F_1(s)}{H(s+)}\text {d}M_{0}(s). \end{aligned}$$

The first term on the right hand side of (7) is a term in an IPCW estimator (Robins 1993) used in the direct binomial regression technique of Scheike et al. (2008) to model all time points. The second term on the right hand side of the equation has expectation zero given the covariates, but may be of importance in reducing the variance when the risk of the 1-event is not small and there is a significant amount of censoring. Evaluating (7) at $F=F_n$ and $X=X_i$, we obtain the infinitesimal jack-knife pseudo-observations,

$$\begin{aligned} \hat{\theta }_{n,i}^{\text {IJ}}&:=\phi (F_n)+\dot{\phi }_{F_n}(X_i) \\&=\int _0^t \frac{1}{\hat{G}_n(s-)}\text {d}N_{1i}(s) +\int _0^t \frac{\hat{F}_{n,1}(t)-\hat{F}_{n,1}(s)}{\hat{S}_n(s)\hat{G}_n(s)}\text {d}\hat{M}_{0i}(s), \end{aligned}$$

where $\hat{M}_{0i}(s)=N_{0i}(s)-\int _0^sY_{\text {c},i}(u)\text {d}\hat{\Lambda }_{n,0}(u)$. The estimates $\hat{F}_{n,1}$, $\hat{S}_n$, $\hat{G}_n$ and $\hat{\Lambda }_{n,0}$ are computed once based on the complete sample.

The R package survival uses a different implementation of infinitesimal jack-knife residuals based on the estimator $\hat{F}_1(t)=\int _0^t\exp \{-\sum _{j=1}^d\hat{\Lambda }_{n,j}(u)\}\text {d}\hat{\Lambda }_{n,1}(u)$ (Terry Therneau, personal communication). Here the infinitesimal jack-knife residuals is based on extending the estimator in a weighted form,

$$\begin{aligned} \hat{F}^w_1(t)=\int _0^t\exp \{-\sum _{j=1}^d\hat{\Lambda }^w_{n,j}(u)\}\text {d}\hat{\Lambda }^w_{n,1}(u), \end{aligned}$$

where $\hat{\Lambda }^w_{n,j}(s)= \int _0^s \{\sum _iw_iY_i(u)\}^{-1}\text {d}\{\sum _i w_iN_{i,j}(u)\}$. The estimator $\hat{F}_1(t)$ is obtained when all $w_i$ are equal to 1/*n*. The infinitesimal jack-knife influence function is obtained by taking the derivative with respects to the weights,

$$\begin{aligned} \frac{\partial \widehat{F}^w_1(t)}{\partial w_i}\vert _{w_i=\frac{1}{n}} \end{aligned}$$

(Jaeckel 1972; Efron 1982).

#### A cohort with left-truncation

In the cohort with left-truncation, the Aalen–Johansen estimate is a functional of the counting process representation, $\frac{1}{n}\sum _{i=1}^n\delta _{X_i}^L$ say, which adjusts for left-truncation by adjusting the at-risk set. Specifically, define the at-risk indicator $Y_{i}^L(s)=1(L_i\le s\le \tilde{T}_i)$, $Y_{\text {c},i}^{L}(s)=1(L_i\le s<\tilde{T}_i)+1(L_i\le s=\tilde{T}_i,\tilde{\Delta }_i=0)$ and counting processes $N_{i,j}^L(s)=1(L_i\le \tilde{T}_i\le s,\tilde{\Delta }_i=j)$. Then we represent the time-to-event data as $\delta _{X_i}^L =(Y_i^L(\cdot ),Y_{\text {c},i}^{L}(\cdot ),N_{i,j}^L(\cdot ),j=0,...,d)^T$. Here $\frac{1}{n}\sum _{i=1}^n\delta _{X_i}^L$ will have limit $F_{|\tilde{T}\ge L}$, say. We let $\hat{G}_n$ and $\hat{F}_{n,j},j=1,\ldots ,d$ denote the Kaplan-Meier estimate of the censoring survival function and the Aalen-Johansen estimate the *j*-event risk, respectively, based on adjusting the at-risk set. The assumption that $C_i$ and $L_i$ are independent is needed to ensure that $\hat{G}_n$ is unbiased. The details are given in Section C of the supplement. More importantly, it is shown in Section C for the special case of the Kaplan–Meier functional, $\chi$ say, that the expectation of its influence function is

$$\begin{aligned} \text {E}_{F_{|\tilde{T}\ge L}}(\dot{\chi }_{F_{|\tilde{T}\ge L}}(X)|Z) =\frac{\text {P}(\tilde{T}\ge L)}{\text {P}(\tilde{T}\ge L|Z)}\{S(t|Z)-S(t)\} . \end{aligned}$$

This will be equal $S(t|Z)-S(t)$ if $\text {P}(\tilde{T}\ge L)=\text {P}(\tilde{T}\ge L|Z)$, i.e., in the case where covariates *Z* do not have any prognostic value (on the distribution of *L*). Thus use of the jack-knife pseudo-observation method for cumulative risk regression in a left-truncated cohort will in general be biased.

The idea of modifying the infinitesimal jack-knife pseudo-observation is to estimate (6), by estimating *F*, using the left-truncated time-to-event data, $F_n:=\frac{1}{n}\sum _{i=1}^n\delta _{X_i}^L$. The estimate of *F* will be written as $\rho (F_n)=\rho _2(\rho _1(F_n))$, where the two mappings

$$\begin{aligned} \rho _1: &F_n \mapsto (\hat{G}_n,\hat{F}_{n,j},j=1,\ldots ,d)^T \\ \rho _2:&(\hat{G}_n,\hat{F}_ {n,j},j=1,\ldots ,d)^T \mapsto (\hat{H}_n,\hat{H}_{n,\text {c}},\hat{H}_{n,j},j=0,\ldots ,d)^T, \end{aligned}$$

and $\hat{S}_n(s)=1-\sum _{j=1}^d\hat{F}_{n,j}(s)$, $\hat{H}_n(s)=\hat{S}_n(s-)\hat{G}_n(s-)$, $\hat{H}_{n,\text {c}}(s)=\hat{S}_n(s)\hat{G}_n(s-)$, $\hat{H}_{n,0}(s)=\int _0^s\hat{S}_n(u)\text {d}(1-\hat{G}_n(u))$ and $\hat{H}_{n,j}(s)=\int _0^s\hat{G}_n(u-)\text {d}\hat{F}_{n,j}(u)$ for $j=1,\ldots ,d$. Inserting $\rho (F_n)$ in (7) results in the estimated infinitesimal jack-knife pseudo-observations

8 $$\begin{aligned} \hat{\theta }_{n,i}^{\text {IJ}}&:=\phi (\rho (F_n))+\dot{\phi }_{\rho (F_n)}(X_i) \\&=\int _0^t \frac{1}{\hat{G}_n(s-)}\text {d}N_{1i}(s) +\int _0^t \frac{\hat{F}_{n,1}(t)-\hat{F}_{n,1}(s)}{\hat{S}_n(s)\hat{G}_n(s)}\text {d}\hat{M}_{0i}(s), \nonumber \end{aligned}$$

where $\hat{M}_{0i}(s)=N_{0i}(s)-\int _0^sY_{\text {c},i}(u)\text {d}\hat{\Lambda }_{n,0}(u)$ is based on the at-risk set $Y_{\text {c},i}$ from the setting without left-truncation. Here all subjects are set to be at risk from time zero. The infinitesimal jack-knife pseudo-observations will not have the correct mean under $\text {P}(\cdot |L_i\le \tilde{T}_i)$, but we can compensate by applying estimated weights, $\hat{w}_i$ say, of the inverse sampling probability, $w_i=F_{L}(\tilde{T}_i)^{-1}$, to the estimating equation,

$$\begin{aligned} \sum _{i=1}^n A(\beta ; Z_i)\hat{w}_i\{\hat{\theta }_{n,i}^{\text {IJ}}-\mu (\beta ; Z_i )\} = 0. \end{aligned}$$

The truncation distribution $F_L(s)=\text {P}(L\le s)$ is estimated by the product-limit estimator of the reversed time, , where $\hat{\Lambda }_{L,n}(s)=\int _0^s\frac{1}{\hat{H}_n(u)}\text {d}N_{L}(u)$ and $N_{L}(s)=\frac{1}{n} \sum _{i=1}^n1(L_i\le s)$. In practice, we will often only be able to estimate $\text {P}(L\le s|L\le \tau )$, for some $\tau >0$, but this is sufficient for the use of sampling weights. The pseudo-observations will satisfy the limiting estimation equation

$$\begin{aligned}&\text {E}\left( A(\beta _0;Z_i)w_i(\phi (F)+\dot{\phi }_F (X_i)-\mu (\beta _0;Z_i))\big |L_i\le \tilde{T}_i,Z_i\right) \\ {}&=\text {E}\left( A(\beta _0;Z_i)w_i1(L_i\le \tilde{T}_i)(\phi (F)+\dot{\phi }_F (X_i)-\mu (\beta _0;Z_i))\big |Z_i\right) /\,\text {P}(L_i\le \tilde{T}_i|Z_i) \\&=\text {E}\left( \text {E}\big ( A(\beta _0;Z_i)w_i1(L_i\le \tilde{T}_i)(\phi (F)+\dot{\phi }_F (X_i)-\mu (\beta _0;Z_i))\big |T_i,C_i,Z_i\big )\big |Z_i\right) /\,\text {P}(L_i\le \tilde{T}_i|Z_i) \\ {}&=\text {E}\left( A(\beta _0;Z_i)(\phi (F)+\dot{\phi }_F (X_i)-\mu (\beta _0;Z_i))\big |Z_i\right) /\,\text {P}(L_i\le \tilde{T}_i|Z_i) \\&=0. \end{aligned}$$

Section D of the supplement shows that the resulting estimate $\hat{\beta }_n$ converges in distribution to a normal distribution with mean zero. A formula for the asymptotic variance is also presented. The variance can be estimated by plug-in of the involved quantities. Since the bootstrap procedure is computationally attractive, we will not proceed further to estimate the asymptotic variance.

## Simulations

Consider a binary exposure covariate, $Z\in \{0,1\}$, with $p_Z=\text {P}(Z=1)$ and a linear model for the event of interest

$$\begin{aligned} F_{1}(s|Z)=(\beta _0+\beta _1 Z)s , s\in [0,1], \end{aligned}$$

and $F_{2}(s|Z)=\eta \cdot s$ for the competing event. The parameter $\beta _0$ is the cumulative risk of 1-events among unexposed subjects ($Z=0$) and $\beta _1$ is the 1-event risk difference between exposed ($Z=1$) and unexposed subjects at time $t=1$. Let *C* be a censoring time independent of events and covariate data. For given $p_{c}\in (0,1]$, we let *C* follow a uniform distribution on the interval $[0,1/p_{c}]$, so that $p_{c}=\text {P}(C_j<1)$. We choose $p_c$ so the observed fraction of censored data before time 1, $p_{\text {oc}}=\text {P}(C<\min (T,1))$ is equal to specified values set below using the relation

$$\begin{aligned} p_{\text {oc}}=\text {P}(C<1)\{\text {P}(T\le 1)/2+\text {P}(T>1)\}. \end{aligned}$$

A similar simulation model was used in Overgaard et al. (2017, 2018); Parner et al. (2020) to evaluate the use of jack-knife pseudo-observations in cohort and case-cohort analyses.

We consider five different scenarios for specific values of $p_Z$, $\beta _0$, $\beta _1$, $\eta$, $p_{\text {oc}}$ to illustrate (1) computational speed in different implementation of the pseudo-observation method; (2) medium and large sample properties of the jack-knife and infinitesimal jack-knife pseudo-observations; (3) comparing the infinitesimal jack-knife pseudo-observation and inverse probability of censoring weighting; (4) medium and large sample properties of infinitesimal jack-knife pseudo-observations in a left-truncated cohort; and (5) comparing the jack-knife and modified infinitesimal jack-knife pseudo-observations in a left-truncated cohort. The computation speed in Scenario 1 was computed as the average of 10 simulations, whereas the simulations in Scenario 2–5 were performed with 10, 000 replications.

**Fig. 1:**
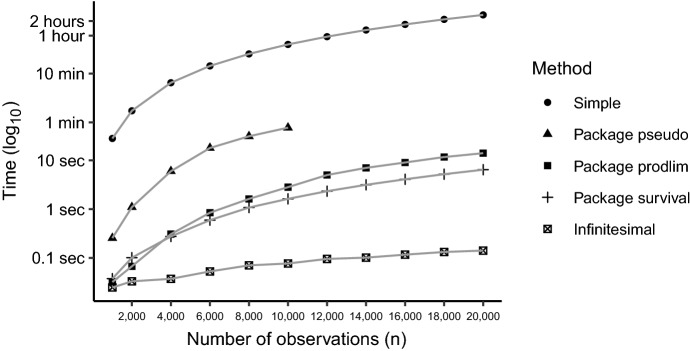
Computational speed in calculating the pseudo-observations on a standard PC. The y-axis uses a log$_{10}$-scale. Infinitesimal is the proposed implementation of infinitesimal jack-knife pseudo-observations

*Scenario 1.* Consider $p_Z=0.5$, unexposed type 1 risk $\beta _0=0.20$, risk difference between exposed and unexposed $\beta _1=0.20$, risk of competing events $\eta =0.20$ and 20% observed censored outcomes $p_{\text {oc}}=0.20$. We compare implementations of the pseudo-observation method in the software program R on a PC with 16 GB ram and Intel Xeon 2.6 GHz processor for sample sizes between $n=1000$ and 20, 000, with 10 replications. The first simple implementation used a package (here the package prodlim) for computing the cumulative incidence for the whole sample and for each leave-one-out samples. This implementation illustrates how pseudo-observations can be calculated using standard statistical software with a loop over all observations, and serves as the reference implementation. The first package in R that presented a more efficient implementation was the package pseudo based on matrix computations. The largest data set that was possible to run using the pseudo package on a PC was with $n=11,000$ observation due to the use of a high dimensional matrix. Another implementation was done in the package prodlim, where much of the coding is in C++ using the R package Cpp. The package survival has an implementation of infinitesimal jack-knife residuals. Finally, an implementation of the proposed infinitesimal jack-knife pseudo-observations. In the set-up used in the current scenario and a sample size of $n=1000$, we found the largest difference between the two implementation of infinitesimal jack-knife observations to be less than $10^{-14}$. The implementation using the package pseudo was only performed for *n* smaller than or equal to 10,000. Compared to the simple implementation, all other implementation were considerably faster (Fig. 1). The proposed implementation of infinitesimal jack-knife pseudo-observations was the fasted implementation.

*Scenario 2.* Consider $p_Z=0.5$, unexposed type 1 risk $\beta _0=0.20$, risk difference between exposed and unexposed $\beta _1=0.10, 0.55$, risk of competing events $\eta =0.20$ and 20% observed censored outcomes $p_{\text {oc}}=0.20, 0.50$. We varied the number of observations $n=100$, 200, 500, 1000 respectively $n=200$, 400, 1000, 2000 for risk difference 0.10 and 0.55, by which we obtained a compatible number of at risk at time 1. In all simulation scenarios, the average number of events was larger than the minimum 10 event per predictor variable suggested in Hansen et al. (2014) for risk differences. The smallest average number of 1-events was in the scenario with $n=100$, $\beta _1=0.10$, $p_{\text {oc}}=0.50$, where the average number of events was 17. Table 1 presents $\sqrt{n}$ times the standard deviation of $\hat{\beta }_1$ based on 10, 000 replications ($\text {SD}_{\text {RD}}$) using both the infinitesimal jack-knife and standard jack-knife pseudo-observations and the average standard error using the Huber–White robust variance estimate (Se$_{\text {HW}}$), the asymptotic variance estimate (Se$_{\Sigma }$) derived from (5) and the bootstrap variance (Se$_{\text {Boot}}$), where the latter was computed with 1000 bootstrap replications. Overall, the variance estimates worked well. However, when the risk difference is large and the censoring rate is moderate, the Huber–White variance estimate is a few percent to high. When both the risk difference is large and the censoring rate is large, the Huber–White variance estimate is too high compared to both the asymptotic and the bootstrap variance. This is in accordance with the simulations and theory of Overgaard et al. (2018).

**Table 1:** Medium and large sample properties of jack-knife and infinitesimal jack-knife pseudo-observations in a cohort without left-truncation

			Jack-knife				Infinitesimal jack-knife			
$p_{\text {oc}}$	$\beta _1$	*n*	Bias	$\sqrt{n}\text {SD}_{\text {RD}}$	$\sqrt{n}\text {Se}_{\text {HW}}$	$\sqrt{n}\text {Se}_{\Sigma }$	Bias	$\sqrt{n}\text {SD}_{\text {RD}}$	$\sqrt{n}\text {Se}_{\text {HW}}$	$\sqrt{n}\text {Se}_{\text {Boot}}$
0.20	0.10	100	0.001	0.942	0.925	0.920	0.001	0.940	0.923	0.925
0.20	0.10	200	0.000	0.927	0.929	0.926	0.000	0.926	0.928	0.928
0.20	0.10	500	0.001	0.920	0.931	0.929	0.001	0.920	0.930	0.930
0.20	0.10	1000	$-$0.000	0.928	0.931	0.930	$-$0.000	0.928	0.931	0.930
0.20	0.55	200	0.001	0.930	0.958	0.917	$-$0.000	0.929	0.956	0.920
0.20	0.55	400	0.000	0.925	0.959	0.919	$-$0.000	0.928	0.959	0.923
0.20	0.55	1000	0.001	0.929	0.959	0.920	0.000	0.932	0.959	0.923
0.20	0.55	2000	0.000	0.913	0.959	0.920	0.000	0.916	0.960	0.924
0.50	0.10	100	0.001	1.139	1.118	1.090	$-$0.000	1.126	1.105	1.095
0.50	0.10	200	0.001	1.128	1.117	1.102	0.001	1.121	1.110	1.104
0.50	0.10	500	$-$0.000	1.130	1.116	1.108	$-$0.000	1.127	1.113	1.109
0.50	0.10	1000	0.000	1.125	1.117	1.113	$-$0.000	1.124	1.116	1.113
0.50	0.55	200	$-$0.001	1.238	1.355	1.192	$-$0.007	1.220	1.335	1.201
0.50	0.55	400	0.001	1.213	1.346	1.199	$-$0.002	1.205	1.337	1.203
0.50	0.55	1000	0.000	1.210	1.339	1.202	$-$0.001	1.207	1.336	1.203
0.50	0.55	2000	$-$0.000	1.219	1.338	1.203	$-$0.001	1.218	1.336	1.203

*Scenario 3.* We now compared the infinitesimal jack-knife pseudo-observations to inverse probability of censoring weighting using only the first part of (7). Considering the unexposed type 1 risk $\beta _0=0.10, 0.20$, the risk difference was $\beta _1=0.05, 0.20, 0.50$, risk of competing events was $\eta =0.20$, the percent of observed censored outcomes was $p_{\text {oc}}=0.10, 0.30, 0.60$ and $n=10,000$. There appears to be an efficiency gain using pseudo-observations as compared to inverse probability of censoring weighting when the risk difference is high and there is a large fraction of censored observations (Table 2). Similarly, in a simulation study, Binder et al. (2014) found a smaller variance of the jack-knife pseudo-observation method when compared to direct binomial regression of Scheike et al. (2008) for risk regression over time.

**Table 2:** Infinitesimal jack-knife pseudo-observation and inverse probability censoring weighting in a cohort without left-truncation. Eff denotes the relative efficiency between the pseudo-observation and IPCW method

			Infinitesimal jack-knife	IPCW	
$p_0$	$\beta _1$	$p_\text {oc}$	$\text {SD}_{\text {RD}}$	$\text {SD}_{\text {RD}}$	Eff
0.10	0.05	0.10	0.0068	0.0068	0.997
0.10	0.05	0.30	0.0074	0.0075	0.990
0.10	0.05	0.60	0.0089	0.0090	0.982
0.10	0.20	0.10	0.0080	0.0081	0.993
0.10	0.20	0.30	0.0087	0.0089	0.981
0.10	0.20	0.60	0.0108	0.0112	0.966
0.10	0.50	0.10	0.0084	0.0087	0.976
0.10	0.50	0.30	0.0095	0.0101	0.938
0.10	0.50	0.60	0.0128	0.0145	0.885
0.20	0.05	0.10	0.0087	0.0087	0.993
0.20	0.05	0.30	0.0095	0.0097	0.979
0.20	0.05	0.60	0.0117	0.0122	0.964
0.20	0.20	0.10	0.0092	0.0093	0.989
0.20	0.20	0.30	0.0102	0.0106	0.967
0.20	0.20	0.60	0.0132	0.0141	0.938
0.20	0.50	0.10	0.0090	0.0093	0.966
0.20	0.50	0.30	0.0102	0.0113	0.905
0.20	0.50	0.60	0.0148	0.0177	0.837

*Scenario 4.* We turn to the medium and large sample properties for the infinitesimal jack-knife pseudo-observation method under left-truncation in the same setting of the complete cohort as in Scenario 1. We considered a left-truncation time with mass 20% at zero and otherwise uniform on the interval (0, 1). The left-truncation time was simulated independently of $(C,T,\Delta ,Z)$. Again, overall both variance estimates worked well (Table 3). Similar to Scenario 2, when the risk difference is high and the censoring rate is moderate the Huber–White variance estimate is a few percent to high, whereas when both the risk difference is high and the censoring rate is high, the Huber–White variance estimate is too high as compared to both the asymptotic and the bootstrap variance.

**Table 3:** Medium and large sample properties of the infinitesimal jack-knife pseudo-observation method in a cohort with left-truncation

			Infinitesimal jack-knife			
$p_{\text {oc}}$	*n*	$\beta _1$	Bias	$\sqrt{n}\text {SD}_{\text {RD}}$	$\sqrt{n}\text {Se}_{\text {HW}}$	$\sqrt{n}\text {Se}_{\text {Boot}}$
0.20	100	0.10	0.001	1.08	1.04	1.05
0.20	200	0.10	0.000	1.07	1.06	1.06
0.20	500	0.10	$-$0.000	1.07	1.07	1.06
0.20	1000	0.10	$-$0.000	1.07	1.07	1.07
0.20	200	0.55	0.000	1.02	1.04	1.00
0.20	400	0.55	$-$0.000	1.00	1.04	1.00
0.20	1000	0.55	$-$0.000	1.01	1.04	1.00
0.20	2000	0.55	0.000	0.99	1.04	1.00
0.50	100	0.10	0.000	1.13	1.13	1.12
0.50	200	0.10	$-$0.000	1.14	1.13	1.13
0.50	500	0.10	$-$0.000	1.15	1.13	1.13
0.50	1000	0.10	$-$0.000	1.13	1.14	1.13
0.50	200	0.55	$-$0.002	1.14	1.25	1.14
0.50	400	0.55	$-$0.002	1.15	1.25	1.14
0.50	1000	0.55	0.000	1.15	1.25	1.13
0.50	2000	0.55	$-$0.001	1.11	1.25	1.13

*Scenario 5.* In this scenario we compared the jack-knife and infinitesimal jack-knife pseudo-observations under left-truncation. We considered covariate distributions $p_Z=0.20,0.50,0.80$, unexposed type-1 risk $\beta _0=0.10,0.20$, risk difference $\beta _1=0.20,0.40,0.60$, risk of competing events $\eta =0.20$, $p_{\text {oc}}=0.20$ in the complete cohort, $n=10,000$ and the same truncation distribution as in Scenario 3. The results in Table 4 showed small bias for the jack-knife pseudo-observations when the covariate distribution is symmetric ($p_Z=0.5$). However, when the covariate distribution is not symmetric a significant bias arose. A symmetric covariate distribution was used in simulations in Grand et al. (2019), Shen (2021) and may explain why the bias was not picked up in their simulations. Due to the large sample size ($n=10,000$) and the high number of replications (10, 000), the infinitesimal jack-knife had the correct average estimate up to 3 decimals in all scenarios.

**Table 4:** Bias of the jack-knife and infinitesimal jack-knife pseudo-observations in a cohort with left-truncation

			Jack-knife	Infinitesimal jack-knife
$p_0$	$\beta _1$	$p_Z$	Ave $\hat{\beta }_1$	Ave $\hat{\beta }_1$
0.10	0.20	0.20	0.210	0.200
0.10	0.20	0.50	0.200	0.200
0.10	0.20	0.80	0.191	0.200
0.10	0.40	0.20	0.443	0.400
0.10	0.40	0.50	0.403	0.400
0.10	0.40	0.80	0.363	0.400
0.10	0.60	0.20	0.707	0.600
0.10	0.60	0.50	0.610	0.600
0.10	0.60	0.80	0.517	0.600
0.20	0.20	0.20	0.211	0.200
0.20	0.20	0.50	0.200	0.200
0.20	0.20	0.80	0.190	0.200
0.20	0.40	0.20	0.445	0.400
0.20	0.40	0.50	0.403	0.400
0.20	0.40	0.80	0.362	0.400
0.20	0.60	0.20	0.711	0.600
0.20	0.60	0.50	0.611	0.600
0.20	0.60	0.80	0.515	0.600

## Example using data on Danish patients with diabetes

**Fig. 2:**
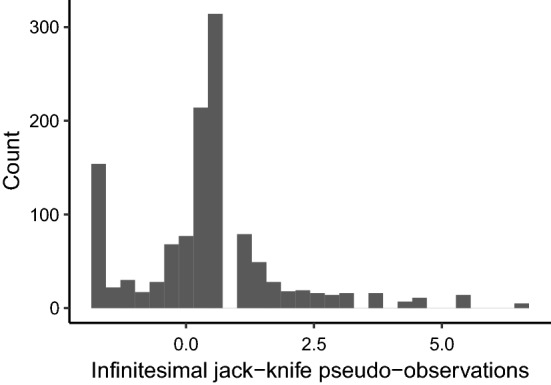
Distribution of the infinitesimal jack-knife pseudo-observations

We used data on Danish patients with diabetes (Green et al. 1981) that was used in Grand et al. (2019) to illustrate the use of standard jack-knife pseudo-observations under left-truncation. Due to the General Data Protection Regulation (GDPR), all time variables registered in years in the current data set were set to integers. From the entire population of the county of Funen in Denmark on July 1, 1973, a total of 1499 were identified with diabetes. The purpose of the study was to estimate mortality risk from the time of diagnosis. Hence, the time scale was time in years from diagnosis until death or censoring (January 1, 1982). The entry time was the time from date of diagnosis until study start (July 1, 1973). Grand et al. (2019) quantified the effect of sex and age at diagnosis on mortality rates using the standard pseudo-observation method and the Cox partial likelihood method. Here, we illustrated the use of infinitesimal jack-knife pseudo-observations to analyse the 30-year cumulative mortality risk for patients diagnosed before 60 years using data on patients with entry before 29 years after diagnosis, leaving 1,216 patients with a median entry of 12 years for analysis. We focused on the comparison of cumulative mortality risk between males and females and compared the results to the standard jack-knife pseudo-observation method. The distribution of the infinitesimal jack-knife pseudo-observations is shown in Fig. 2. The presented Wald confidence intervals and statistical tests are computed using the Huber-White variance.

The infinitesimal jack-knife pseudo-observation method estimated a 30-year mortality risk for females of 59.5% (52.6$-$67.2%) and a relative risk for males compared to females of 0.96 (0.82$-$1.13). The results are similar to estimates from the standard jack-knife pseudo-observation method of 59.9% (95% CI 53.5$-$67.1%) and 0.99 (0.85$-$1.16) and in agreement with the Aalen–Johansen estimates of the 30-year mortality risk for females of 60.2% (95% CI 53.7$-$66.6%) and for males of 59.7% (52.7$-$66.7%). Including sex and age at diagnosis as a continuous variable in a log-linear model resulted in the estimates in Table 5. The baseline risk of females and the age at diagnosis relative risks were in agreement between the two methods, but some difference was seen in the female/male relative risk, which may be due to the bias in the standard jack-knife pseudo-observation method.

**Table 5:** Baseline risk for females diagnosed at 40 years and relative risk (RR) for males compared to females and one-year difference in age at diagnosis using the infinitesimal jack-knife and jack-knife pseudo-observation method

	Infinitesimal jack-knife			Jack-knife	
	Estimate	*P* value		Estimate	*P* value
Females (risk)	0.65 (0.57$-$0.72)	<0.001		0.65 (0.58$-$0.72)	<0.001
Males vs. females (RR)	1.03 (0.90$-$1.19)	0.64		1.16 (0.98$-$1.36)	0.078
Age at diagnosis (RR)	1.04 (1.03$-$1.04)	<0.001		1.03 (1.03$-$1.04)	<0.001

## Discussion

We suggested using infinitesimal jack-knife pseudo-observations for regression analysis and showed that they are asymptotically equivalent to jack-knife pseudo-observations. The infinitesimal jack-knife pseudo-observations are faster to compute, making the non-parametric bootstrap attractive for variance estimation. We explained why the core assumption on the influence function of the base estimate is needed for unbiased inference. We then showed that the condition on the influence function of the Kaplan–Meier base estimate is not satisfied for a left-truncated cohort and presented a modification of the infinitesimal jack-knife pseudo-observations that together with sampling weights applied to the estimating equation yielding unbiased estimates of the regression parameters. Similar infinitesimal jack-knife pseudo-observations were implemented for the Kaplan–Meier estimate, the Aalen–Johansen estimate and general multi-state models in the R package survival (Therneau 2021). With left-truncation, the package currently computes the infinitesimal jack-knife pseudo-observations and not the proposed modified infinitesimal jack-knife pseudo-observations. The example of a left-truncated cohort illustrates that the condition on the influence function of the base estimate limits the base estimates that can be used with the pseudo-observation method. We conjecture that the condition on the influence function may not be satisfied in other applications of pseudo-observations described in the literature.

The pseudo-observation method in the presented basic form assumes that the censoring is independent of the time-to-event data and covariates. The method can be modified to censoring that depending on covariates by stratifying the calculation of the pseudo-observations (Andersen and Pohar Perme 2010) or using a regression model for the censoring event rate (Binder et al. 2014; Xiang and Murray 2012; Overgaard et al. 2019).

A central property of the proposed modification of the infinitesimal jack-knife pseudo-observation method is that it avoids modelling the truncation probability P$(v\le T|Z)$, for *v* smaller than the time point of interest *t*, which is necessary in the approach of Li and Peng (2014). The proposed method separates the modelling of the event distribution and the left-truncation distribution. This is similar to the approach in Zhang et al. (2011), where weights were applied to elements in the score equation for the Fine–Gray subdistribution hazard regression model. A similar approach for the Fine–Gray regression model was used in Geskus (2011) and for direct regression models for the restricted mean survival time in Cortese et al. (2017) using inverse probability weighting of both censoring and left-truncation. Both the approach of Geskus (2011) and Cortese et al. (2017) do not assume independence of the truncation time and censoring time. It is a limitation of the proposed infinitesimal jack-knife pseudo-observation method that the truncation time and censoring time are assumed to be independent.

## Supplementary Information

Below is the link to the electronic supplementary material.

Supplementary file 1 (pdf 303 KB)

## Declaration

### Supplementary Materials

The web supplement Section A includes details on functional differentiability; Section B shows proof of the asymptotic equivalence of the jack-knife pseudo-observations and the infinitesimal jack-knife pseudo-observation; Section C shows theorem and proof of the asymptotic distribution when using the modified infinitesimal pseudo-observations in (8); Section D shows computation of the expectation of the Kaplan–Meier influence function with left-truncation.
