# Supplementary material for: Regression models for censored time-to-event data using infinitesimal jack-knife pseudo-observations, with applications to left-truncation
Source: Lifetime Data Anal. 2023 May 8;29(3):654–71. doi: 10.1007/s10985-023-09597-5 (PMC10258172; doi:10.1007/s10985-023-09597-5)
Supplement: Supplementary file 1 — (pdf 303 KB) [file 10985_2023_9597_MOESM1_ESM.pdf]

Regression models for censored time-to-event data using infinitesimal jack-knife pseudo-observations, with applications to left-truncation

Supplementary material

A: Differentiability of functionals, p-variation and influence functions

The following presents a review of differentiability in Banach spaces, with special focus on the function space of bounded p-variation. Further, definitions and properties of the first and second order influence functions are also presented. The description is based on Dudley and Norvaiša (1999, 2011). A similar review was presented in the supplement of Overgaard et al. (2017).

Differentiability of functionals

Consider a functional $\phi : W \to \mathbf{E}$ defined on a open subset $W \subseteq \mathbf{D}$, where $\mathbf{D}$ and $\mathbf{E}$ are Banach spaces. The following is based on Fréchet differentiability, which is one of the stronger types of differentiability with the important properties that differentiability implies continuity and that the chain rule applies. A functional ϕ is differentiable at $f \in W$ if there exists a continuous, linear map $\phi'_f : \mathbf{D} \to \mathbf{E}$ so that

$$\parallel \phi(f+h) - \phi(f) - \phi'_f(h) \parallel_{\mathbf{E}} = o(\parallel h \parallel_{\mathbf{D}}),$$

where $\phi'_f(h)$ is called the derivative of ϕ at f in direction h. The derivative $f \mapsto \phi'_f$ is a functional from W into $L^1(\mathbf{D}, \mathbf{E})$, the space of linear, continuous maps from $\mathbf{D}$ to $\mathbf{E}$. The space $L^1(\mathbf{D}, \mathbf{E})$ is a Banach space when equipped with the operator norm: For a continuous linear map $a : \mathbf{D} \to \mathbf{E}$ there exists a constant $K > 0$ so that $\parallel a(f) \parallel_{\mathbf{D}} \leq K \parallel f \parallel_{\mathbf{E}}$ for all $f \in \mathbf{D}$. The operator norm of a is the smallest of such constants,

$$\parallel a \parallel_{\text{op}} = \inf\{K > 0 : \parallel a(f) \parallel_{\mathbf{E}} \leq K \parallel f \parallel_{\mathbf{D}}, \text{ for all } f \in \mathbf{D}\}.$$

Continuity and differentiability of $f \mapsto \phi'_f$ is defined in $L^1(\mathbf{D}, \mathbf{E})$ equipped with the operator norm. A functional ϕ is continuously differentiable, or C^1, if it is differentiable and the derivative ϕ' is continuous with respect to the operator norm. Higher order differentiability can be defined recursively for $k > 1$ on the space

$$L^k(\mathbf{D}, \mathbf{E}) = L^1(\mathbf{D}, L^{k-1}(\mathbf{D}, \mathbf{E})),$$

with the operator norm

$$\parallel a \parallel_{\text{op}} = \inf\{K > 0 : \parallel a(f_1, \ldots, f_k) \parallel_{\mathbf{E}} \leq K \parallel f_1 \parallel_{\mathbf{D}} \cdots \parallel f_k \parallel_{\mathbf{D}}, \text{ for all } f_i \in \mathbf{D}\}.$$

The Schwarz Theorem states that the k'th derivative $\phi_f^{(k)}$ is symmetric in its argument, cf. Theorem 5.27 of Dudley and Norvaiša (2011).

A functional $\phi : W \to \mathbf{E}$ is analytic if it has a Taylor expansion around each point of W. Analyticity of ϕ on W implies that ϕ is C^k on W for any $k \geq 1$, cf. Theorem 5.28 of Dudley

and Norvaiša (2011). When ϕ is a C^k functional, we have the kth order Taylor expansion with an integral remainder

$$\phi(f+g) = \sum_{j=0}^{k} \frac{1}{j!}\phi_f^{(j)}(g,\dots,g) + \frac{1}{(k-1)!}\int_0^1 (1-s)^{k-1}(\phi_{f+sg}^{(k)} - \phi_f^{(k)})(g,\dots,g)\mathrm{d}s, \quad (1)$$

if $f + sg \in W$ for all $s \in [0,1]$, where $\phi_f^{(0)} = \phi(f)$, cf. Theorem 5.42 of Dudley and Norvaiša (2011). If $\phi : W \to \mathbf{E}$ and $\psi : V \to \mathbf{F}$ are C^k then the composition $\psi \circ \phi$ is also C^k, cf. Theorem 2.10.0 of Keller (2006). The chain rule specifies that the derivative of $\psi \circ \phi$ at f in direction g is

$$(\psi \circ \phi)'_f(g) = \psi'_{\phi(f)}(\phi'_f(g)).$$

A functional $\phi : W \to \mathbf{E}$ is said to be Lipschitz continuous if there exists a constant $K > 0$ so that

$$\| \phi(f) - \phi(g) \|_{\mathbf{E}} \le K \| f - g \|_{\mathbf{D}},$$

for all $f, g \in W$. The functional is said to be locally Lipschitz continuous if for all $f \in W$ there exists a ball, $B_f \subseteq W$, around f and a constant $K_f > 0$ so that

$$\| \phi(g) - \phi(h) \|_{\mathbf{E}} \le K_f \| g - h \|_{\mathbf{D}},$$

for all $g, h \in B_f$. If a functional $\phi : W \to \mathbf{E}$ is continuously differentiable (C^1) then it is also locally Lipschitz continuous, cf. Proposition 1.4 in the supplement of Overgaard et al. (2017). We are particularly interested in the case with functional in C^2 with locally Lipschitz continuous second order derivatives. For two C^2 functions, $\phi : W \to \mathbf{E}$ and $\psi : V \to \mathbf{F}$, if both ϕ and ψ have locally Lipschitz continuous second order derivatives, then $\psi \circ \phi$ also has a locally Lipschitz continuous second order derivative, cf. Proposition 1.5 in the supplement of Overgaard et al. (2017).

Functions with finite p-variation

Let $(\Omega, \mathcal{F}, \mathrm{P})$ be a probability space, $(\mathcal{X}, \mathcal{A})$ a measure space and $(\mathbf{D}, \| \cdot \|_{\mathbf{D}})$ a Banach space. Consider an i.i.d. sample $X_1, \dots, X_n$ defined on $(\Omega, \mathcal{F}, \mathrm{P})$ with values in $\mathcal{X}$ and a map $\delta_{(\cdot)}$: $\mathcal{X} \to \mathbf{D}$. Let $F_n = \frac{1}{n}\sum_{i=1}^n \delta_{X_i}$ be a sample average and let F denote the limit of F_n when it exists. We consider base estimates $\hat\theta_n$ which are functional of a sample average, $\hat\theta_n = \phi(F_n)$. The functional ϕ can be considered a map from $\mathbf{D}$ into a parameter Banach space, $\mathbf{E}$ say, where the parameter space is $\mathbb{R}$ with the Euclidean norm in our applications.

The Banach space $(\mathbf{D}, \| \cdot \|_{\mathbf{D}})$ will be the space of bounded functions in p-variation. The p-variation of a function $f : J \to R$ on the interval $J \subseteq \mathbb{R}$ is defined by

$$v_p(f; J) = \sup \sum_{i=1}^{m} |f(x_{i-1}) - f(x_i)|^p,$$

where the supremum is taken over all $m \in \mathbb{N}$ and points $x_0 < x_1 < \dots < x_m$ in the interval J. The space of functions of bounded p-variation

$$\mathcal{W}_p = \{ f : J \to \mathbb{R} \mid v_p(f) < \infty \}$$

with the p-variation norm

$$\| f \|_{[p]} = v_p(f; J)^{\frac{1}{p}} + \| f \|_\infty$$

is a Banach space (Dudley and Norvaiša, 2011).

We are interested in the rate of convergence of $F_n = \frac{1}{n}\sum_{i=1}^n \delta_{X_i}$. The results of Dudley and Norvaiša (2011), Theorem 6.2 of Part I, deals with F_n that are the empirical distribution functions of a one-dimensional i.i.d. sample with common distribution function F:

$$\| F_n - F \|_{[p]} = O_{\mathrm{P}}(n^{\frac{1-p}{p}}(\log\log n)^{\frac{1}{2}}), \tag{2}$$

for $1 \le p < 2$. The result was extended to δ_x that is a vector of at-risk and counting processes with at most one jump, with the same rate as in (2), in the supplement of Overgaard et al. (2017), Lemma 2.1. In particular, the rate of convergence $\| F_n - F \|_{[p]} = o_{\mathrm{P}}(n^{-1/4})$ for $p \in [\frac{4}{3}, 2)$ is sufficient for our application. We consider in the present paper base functional ϕ that are differentiable, and indeed analytic, for $1 \le p < 2$. This is proven by considering the base estimate functional as composition of multiplication, integration and product integration.

Influence functions

Consider a base estimate of the form $\hat{\theta}_n = \phi(F_n)$, with $F_n = \frac{1}{n}\sum_{i=1}^n \delta_{X_i}$. When ϕ is differentiable at F, a first order Taylor expansion gives

$$\begin{aligned}\phi(F_n) &= \phi(F) + \phi'_F(F_n - F) + O_{\mathrm{P}}(\| F_n - F \|)\\ &= \phi(F) + \frac{1}{n}\sum_{i=1}^n \phi'_F(\delta_{X_i} - F) + O_{\mathrm{P}}(\| F_n - F \|).\end{aligned}$$

The functional $\dot{\phi}_F(X_i) := \phi'_F(\delta_{X_i} - F)$ is called the (first order) influence function of ϕ. When ϕ has locally Lipschitz derivative, the above convergence can be strengthened to

$$\phi(F_n) = \phi(F) + \frac{1}{n}\sum_{i=1}^n \phi'_F(\delta_{X_i} - F) + O_{\mathrm{P}}(\| F_n - F \|^2).$$

If F_n converge to F with the rate in (2), we then have

$$\phi(F_n) = \phi(F) + \frac{1}{n}\sum_{i=1}^n \dot{\phi}_F(X_i) + o_{\mathrm{P}}(n^{-\frac{1}{2}}). \tag{3}$$

Similarly, the functional $\ddot{\phi}_F(X_i, X_j) = \phi'_F(\delta_{X_i} - F, \delta_{X_j} - F)$ is called the second order influence function of ϕ. The first and second order influence function satisfies

$$\mathrm{E}(\dot{\phi}_F(X)) = 0 \text{ and } \mathrm{E}(\ddot{\phi}_F(X, x)) = 0 \text{ for all } x \in \mathcal{X}, \tag{4}$$

cf. formula (3.23) and (3.24) in Overgaard et al. (2017).

B: Infinitesimal jack-knife pseudo-observations

Let $(X_1, Z_1), \dots, (X_n, Z_n)$ denote an i.i.d. sample of observations with time-to-event data X_i and covariate Z_i. Consider a base estimate ϕ that is a functional of averages of the observations $F_n = \frac{1}{n}\sum_{i=1}^n \delta_{X_i}$. Let F denote the limit of F_n when it exists. Define the jack-knife pseudo-observations,

$$\hat{\theta}_{n,i} = n\hat{\theta}_n - (n-1)\hat{\theta}_n^{(i)}. \tag{5}$$

and the infinitesimal jack-knife pseudo-observations,

$$\hat{\theta}^{\mathrm{IJ}}_{n,i} = \phi(F_n) + \dot{\phi}_{F_n}(X_i). \tag{6}$$

We let $\| \cdot \|$ denote the p-variation norm from the previous section.

Theorem 1. *Assume that $\| F_n - F \| = o_P(n^{-\frac{1}{4}})$ and that $\| \delta_x \| \le c$. Let ϕ be two times differentiable with a locally Lipschitz continuous second order derivative, e.g. three times continuous differentiable. Then $\hat{\theta}_{n,i} - \hat{\theta}^{IJ}_{n,i} = o_P(n^{-1/2})$, uniformly in $i = 1, ..., n$.*

Proof. The infinitesimal jack-knife pseudo-observation for observation x is defined in terms of the function

$$\psi(f) := \phi(f) + \phi'_f(\delta_x - f),$$

evaluating at $f = F_n$. The function ψ is differentiable with first order derivative at f in direction g,

$$\begin{aligned}\psi'_f(g) &= \phi'_f(g) + \phi''_f(\delta_x - f, g) + \phi'_f(-g)\\ &= \phi''_f(\delta_x - f, g).\end{aligned}$$

The function ψ satisfies the first order Taylor expansion with integral remainder,

$$\begin{aligned}\psi(f+g) &= \psi(f) + \psi'_f(g) + \int_0^1 (\psi'_{f+sg} - \psi'_f)(g)\mathrm{d}s\\ &= \phi(f) + \phi'_f(\delta_x - f) + \phi''_f(\delta_x - f, g) + \int_0^1 (\psi'_{f+sg} - \psi'_f)(g)\mathrm{d}s,\end{aligned}$$

cf. (1). Consider the integrand in the remainder,

$$\begin{aligned}&\| (\psi'_{f+sg} - \psi'_f)(g) \|\\ =&\| \phi''_{f+sg}(\delta_x - (f+sg), g) - \phi''_f(\delta_x - f, g) \|\\ =&\| (\phi''_{f+sg} - \phi''_f)(\delta_x - f, g) - s\phi''_{f+sg}(g,g) \|\\ \leq&\| \phi''_{f+sg} - \phi''_f \|_{\mathrm{op}} \cdot \| \delta_x - f \| \cdot \| g \| + \| \phi''_{f+sg} \|_{\mathrm{op}} \cdot \| g \| \cdot \| g \| .\end{aligned}$$

Since ϕ''_f is locally Lipschitz, it is possible to find a constant $K_f > 0$ so that $\| \phi''_{f+sg} - \phi''_f \|_{\mathrm{op}} \leq sK_f \| g \|$ when $\| g \|$ is small. With the same constant, we can also bound

$$\| \phi''_{f+sg} \|_{\mathrm{op}} \leq \| \phi''_{f+sg} - \phi''_f \|_{\mathrm{op}} + \| \phi''_f \|_{\mathrm{op}} \leq sK_f \| g \| + \| \phi''_f \|_{\mathrm{op}} .$$

In total, the integrand in the remainder is dominated by

$$\begin{aligned}&\| (\psi'_{f+sg} - \psi'_f)(g) \|\\ &\leq sK_f \| g \| \cdot (c + \| f \|) \cdot \| g \| + (sK_f \| g \| + \| \phi''_f \|_{\mathrm{op}}) \cdot \| g \| \cdot \| g \|\\ &= O(\| g \|^2),\end{aligned}$$

uniformly in x for fixed f. Evaluated at $f = F$, $g = F_n - F$, where $F_n - F$ is small with large probability when n is large,

$$\phi(F_n) + \phi'_{F_n}(\delta_{X_i} - F_n) = \phi(F) + \phi'_F(\delta_{X_i} - F) + \phi''_F(\delta_{X_i} - F, F_n - F) + O_{\mathrm{P}}(\| F_n - F \|^2).$$

As the second order derivative is locally Lipschitz continuous, there exists a constant $K > 0$ so that for large n with high probability

$$\begin{aligned}&\sqrt{n}\Big|\frac{1}{n}\sum_{j=1}^{n} \ddot{\phi}_F(X_i, X_j) - \frac{1}{n-1}\sum_{j\neq i}^{n} \ddot{\phi}_F(X_i, X_j)\Big| \qquad (7)\\ &\leq \Big|\sqrt{n}(1 - \frac{n}{n-1})[\frac{1}{n}\sum_{j=1}^{n} \ddot{\phi}_F(X_i, X_j)] + \sqrt{n}\frac{1}{n-1}\ddot{\phi}_F(X_i, X_i)\Big|\\ &\leq \frac{\sqrt{n}}{n-1}K(c + \| F \|) \cdot \| F_n - F \| + \frac{\sqrt{n}}{n-1}K(c + \| F \|)^2.\end{aligned}$$

The right-hand side of (7) converge to zero as $n \to \infty$. Using the assumption that $\| F_n - F \| = o_{\mathrm{P}}(n^{-\frac{1}{4}})$, we therefore obtain the approximation of the infinitesimal jack-knife pseudo-

observations,

$$\begin{aligned}\hat{\theta}^{\mathrm{IJ}}_{n,i} &= \phi(F_n) + \phi'_{F_n}(\delta_{X_i} - F_n)\\ &= \phi(F) + \dot{\phi}_F(X_i) + \frac{1}{n}\sum_{j=1}^{n}\ddot{\phi}_F(X_i, X_j) + o_{\mathrm{P}}(n^{-\frac{1}{2}})\\ &= \phi(F) + \dot{\phi}_F(X_i) + \frac{1}{n-1}\sum_{j\neq i}^{n}\ddot{\phi}_F(X_i, X_j) + o_{\mathrm{P}}(n^{-\frac{1}{2}})\\ &= \hat{\theta}_{n,i} + o_{\mathrm{P}}(n^{-\frac{1}{2}}),\end{aligned}$$

uniformly in $i = 1, \dots, n$. In the last equation, we have used Proposition 3.1 in Overgaard et al. (2017) in the approximation of $\hat{\theta}_{n,i}$. □

C: Competing risk data with left-truncation

Define the modified at-risk indicators $Y_i^L(s) = 1(L_i \le s \le \tilde{T}_i)$, $Y_{\mathrm{c},i}^L(s) = 1(L_i \le s < \tilde{T}_i) + 1(L_i \le s = \tilde{T}_i, \tilde{\Delta}_i = 0)$ and counting processes $N_{i,j}^L(s) = 1(L_i \le \tilde{T}_i \le s, \tilde{\Delta}_i = j)$ with expectations $H_{|\tilde{T}\ge L}(s) = \mathrm{E}(Y_i^L(s)|\tilde{T} \ge L)$, $H_{\mathrm{c}|\tilde{T}\ge L}(s) = \mathrm{E}(Y_{\mathrm{c},i}^L(s)|\tilde{T} \ge L)$ and $H_{j|\tilde{T}\ge L}(s) = \mathrm{E}(N_{ij}^L(s)|\tilde{T} \ge L)$. The empirical versions are $\hat{H}_{n|\tilde{T}\ge L}(s) = \frac{1}{n}\sum_{i=1}^n Y_i^L(s)$, $\hat{H}_{n,\mathrm{c}|\tilde{T}\ge L}(s) = \frac{1}{n}\sum_{i=1}^n Y_{\mathrm{c},i}^L(s)$ and $\hat{H}_{n,j|\tilde{T}\ge L}(s) = \frac{1}{n}\sum_{i=1}^n N_{i,j}^L(s)$. The combined sets of functions are denoted

$$\begin{aligned}F_{|\tilde{T}\ge L} &= (H_{|\tilde{T}\ge L}, H_{\mathrm{c}|\tilde{T}\ge L}, H_{j|\tilde{T}\ge L}, j = 0, ..., d)^T\\ \delta_{X_i}^L &= (Y_i^L(\cdot), Y_{\mathrm{c},i}^L(\cdot), N_{i,j}^L(\cdot), j = 0, ..., d)^T.\end{aligned}$$

Assume that $\mathrm{P}(L \le s \le C)$ is bounded away from zero on $[0, t]$. Using the independence of (T, Δ), C and L, we have

$$\int_0^s \frac{1}{H_{|\tilde{T}\ge L}(u)} H_{j|\tilde{T}\ge L}(\mathrm{d}u) = \Lambda_j(s)$$

for $j = 1, \dots, d$, and similarly for the censoring. The integrated intensity, survival function and 1-event cumulative incidence can be estimated by $\hat{\Lambda}_{n,j}(s) = \int_0^s \frac{1}{\hat{H}_{n|\tilde{T}\ge L}(u)} \mathrm{d}\hat{H}_{n,j|\tilde{T}\ge L}(u)$, $j = 1, \dots, d$, $\hat{\Lambda}_{n,0}(s) = \int_0^s \frac{1}{\hat{H}_{n,\mathrm{c}|\tilde{T}\ge L}(u)} \mathrm{d}\hat{H}_{n,0|\tilde{T}\ge L}(u)$, $\hat{S}_n(s) = \prod_0^s\{1 - \mathrm{d}\hat{\Lambda}_{n,\cdot}(u)\}$ and $\hat{F}_{n,1}(s) = \int_0^s \hat{S}_n(u-)\mathrm{d}\hat{\Lambda}_{n,1}(u)$.

For the condition on the influence function, we consider for simplicity the scenario without competing risk, so that one minus the Aalen–Johansen estimate reduces to the Kaplan–Meier estimate. Let χ denote the Kaplan–Meier functional of $\frac{1}{n}\sum_{i=1}^n \delta_{X_i}^L$. With a similar argument as in Overgaard et al. (2017), the Kaplan–Meier influence function in the sampled cohort is

$$\dot{\chi}_{F_{|\tilde{T}\ge L}}(X) = -S(t)\int_0^t \frac{1}{(1 - \Delta\Lambda_1(s))H_{|\tilde{T}\ge L}(s)}\{N_1^L(\mathrm{d}s) - Y^L(s)\Lambda_1(\mathrm{d}s)\}, \tag{8}$$

with expectation

$$\begin{aligned}&\mathrm{E}_{F_{|\tilde{T}\ge L}}(\dot{\chi}_{F_{|\tilde{T}\ge L}}(X)|Z)\\ &= -S(t)\{\int_0^t \frac{H_{|\tilde{T}\ge L}(s|Z)}{(1 - \Delta\Lambda_1(s))H_{|\tilde{T}\ge L}(s)}\Lambda_1(\mathrm{d}s|Z) - \int_0^t \frac{H_{|\tilde{T}\ge L}(s|Z)}{(1 - \Delta\Lambda_1(s))H_{|\tilde{T}\ge L}(s)}\Lambda_1(\mathrm{d}s)\}\\ &= -S(t)\frac{\mathrm{P}(\tilde{T} \ge L)}{\mathrm{P}(\tilde{T} \ge L|Z)}\int_0^t \frac{S(s-|Z)}{(1 - \Delta\Lambda_1(s))S(s-)}\{\Lambda_1(\mathrm{d}s|Z) - \Lambda_1(\mathrm{d}s)\}\\ &= \frac{\mathrm{P}(\tilde{T} \ge L)}{\mathrm{P}(\tilde{T} \ge L|Z)}\int_0^t \prod_0^{s-}(1 - \Lambda_1(\mathrm{d}u|Z))\{\Lambda_1(\mathrm{d}s) - \Lambda_1(\mathrm{d}s|Z)\}\prod_s^t(1 - \Lambda_1(\mathrm{d}u))\\ &= \frac{\mathrm{P}(\tilde{T} \ge L)}{\mathrm{P}(\tilde{T} \ge L|Z)}\{S(t|Z) - S(t)\},\end{aligned}$$

where the last equation comes from the Duhamel equation (Johansen and Gill, 1990).

D: Modified infinitesimal jack-knife pseudo-observations

Let $\bar{X}_1 = (X_1, Z_1), \ldots, \bar{X}_n = (X_n, Z_n)$ denote an i.i.d. sample of observations with time-to-event data X_i and covariate Z_i. Consider modified infinitesimal jack-knife pseudo-observations of the form

$$\hat{\theta}^{\mathrm{IJ}}_{n,i} = \phi(\rho(F_n)) + \phi'_{\rho(F_n)}(\delta^*_{X_i} - \rho(F_n)), \tag{9}$$

where $F_n = \frac{1}{n}\sum_{i=1}^n \delta_{X_i}$ and $\delta^*_{X_i}$ is map of X_i. We let F denote the limit of F_n when it exists. In the application to a cohort with left-truncation, F corresponds to the measure conditional on truncation. Formula (9) can be seem as an estimate of the infinitesimal jack-knife pseudo-observations in (6). The following result extend Theorem 1 to modified infinitesimal jack-knife pseudo-observations.

Proposition 1. *Assume that $\| F_n - F \| = o_P(n^{-\frac{1}{4}})$ and that $\| \delta_x \|, \| \delta^*_x \| \leq c$. Let the base estimate function ϕ be two times differentiable with a locally Lipschitz continuous second order derivative, e.g. three times continuous differentiable. Let ρ be differentiable with a locally Lipschitz continuous derivative, e.g. two times continuous differentiable. Then*

$$\begin{aligned}\hat{\theta}^{IJ}_{n,i} = \phi(\rho(F)) + \phi'_{\rho(F)}(\delta^*_{X_i} - \rho(F)) + \frac{1}{n}\sum_{j=1}^n \phi''_{\rho(F)}(\delta^*_{X_i} - \rho(F), \rho'_F(\delta_{X_j} - F)) \\ + O_P(\| F_n - F \|^2).\end{aligned}$$

Proof. The infinitesimal jack-knife pseudo-observation for observation x is defined by the function

$$\psi(f) := \phi(\rho(f)) + \phi'_{\rho(f)}(\delta^*_x - \rho(f)),$$

evaluating at $f = F_n$. The function ψ is differentiable with first order derivative at f in direction g,

$$\begin{aligned}\psi'_f(g) &= \phi'_{\rho(f)}(\rho'_f(g)) + \phi''_{\rho(f)}(\delta^*_x - \rho(f), \rho'_f(g)) + \phi'_{\rho(f)}(-\rho'_f(g)) \\ &= \phi''_{\rho(f)}(\delta^*_x - \rho(f), \rho'_f(g)).\end{aligned}$$

The function ψ satisfies the first order Taylor expansion with integral remainder,

$$\begin{aligned}\psi(f+g) &= \psi(f) + \psi'_f(g) + \int_0^1 (\psi'_{f+sg} - \psi'_f)(g)\mathrm{d}s \\ &= \phi(\rho(f)) + \phi'_{\rho(f)}(\delta^*_x - \rho(f)) + \phi''_{\rho(f)}(\delta^*_x - \rho(f), \rho'_f(g)) + \int_0^1 (\psi'_{f+sg} - \psi'_f)(g)\mathrm{d}s,\end{aligned}$$

cf. (1). Consider the integrand in the remainder,

$$\begin{aligned}&\| (\psi'_{f+sg} - \psi'_f)(g) \| \\ &= \| \phi''_{\rho(f+sg)}(\delta^*_x - \rho(f+sg), \rho'_{f+sg}(g)) - \phi''_{\rho(f)}(\delta^*_x - \rho(f), \rho'_f(g)) \| \\ &= \| (\phi''_{\rho(f+sg)} - \phi''_{\rho(f)})(\delta^*_x - \rho(f), \rho'_f(g)) - \phi''_{\rho(f+sg)}(\rho(f+sg) - \rho(f), \rho'_{f+sg}(g)) \| \\ &\leq \| \phi''_{\rho(f+sg)} - \phi''_{\rho(f)} \|_{\mathrm{op}} \cdot \| \delta^*_x - \rho(f) \| \cdot \| \rho'_f \|_{\mathrm{op}} \cdot \| g \| \\ &\quad + \| \phi''_{\rho(f+sg)} \|_{\mathrm{op}} \cdot \| \rho(f+sg) - \rho(f) \| \cdot \| \rho'_{f+sg} \|_{\mathrm{op}} \cdot \| g \| .\end{aligned}$$

Since $\phi''_{\rho(f)}$ is locally Lipschitz, it is possible to find a constant $K_f > 0$ so that

$$\| \phi''_{\rho(f+sg)} - \phi''_{\rho(f)} \|_{\mathrm{op}} \leq sK_f \| g \|$$

when $\| g \|$ is small. With the same constant, we can also bound

$$\| \phi''_{\rho(f+sg)} \|_{\mathrm{op}} \leq \| \phi''_{\rho(f+sg)} - \phi''_{\rho(f)} \|_{\mathrm{op}} + \| \phi''_{\rho(f)} \|_{\mathrm{op}} \leq sK_f \| g \| + \| \phi''_f \|_{\mathrm{op}} .$$

Since ρ is differentiable with locally Lipschitz derivative, we can find a constant $\bar{K}_f > 0$ so that $\| \rho(f + sg) - \rho(f) \| \leq s\bar{K}_f \| g \|$ when $\| g \|$ is small. Finally, since ρ'_f is locally Lipschitz, it is possible to find a constant $\tilde{K}_f > 0$ so that

$$\| \rho'_{f+sg} \|_{\text{op}} \leq \| \rho'_{f+sg} - \rho'_f \|_{\text{op}} + \| \rho'_f \|_{\text{op}} \leq s\tilde{K}_f \| g \| + \| \rho'_f \|_{\text{op}} .$$

In total, the integrand in the remainder is dominated by

$$\begin{aligned}
&\| (\psi'_{f+sg} - \psi'_f)(g) \| \\
&\leq sK_f \| g \| \cdot (c + \| \rho(f) \|) \cdot \| \rho'_f \|_{\text{op}} \cdot \| g \| \\
&\quad + (sK_f \| g \| + \| \phi''_f \|_{\text{op}}) \cdot \bar{K}_f \| g \| \cdot (s\tilde{K}_f \| g \| + \| \rho'_f \|_{\text{op}}) \cdot \| g \| \\
&= o(\| g \|^2),
\end{aligned}$$

uniformly in x for fixed f. Evaluated at $f = F$, $g = F_n - F$, where $F_n - F$ is small with large probability when n is large,

$$\begin{aligned}
&\phi(\rho(F_n)) + \phi'_{\rho(F_n)}(\delta^*_{X_i} - \rho(F_n)) \\
&= \phi(\rho(F)) + \phi'_{\rho(F)}(\delta^*_{X_i} - \rho(F)) + \phi''_{\rho(F)}(\delta^*_{X_i} - \rho(F), \rho'_F(F_n - F)) + O_{\text{P}}(\| F_n - F \|^2) \\
&= \phi(\rho(F)) + \phi'_{\rho(F)}(\delta^*_{X_i} - \rho(F)) + \frac{1}{n} \sum_{j=1}^{n} \phi''_{\rho(F)}(\delta^*_{X_i} - \rho(F), \rho'_F(\delta_{X_j} - F)) \\
&\quad + O_{\text{P}}(\| F_n - F \|^2).
\end{aligned}$$

□

Let $\omega_{F_n}(\cdot)$ be an estimated weight function and $A(\beta; Z_i)$ a column vector. Consider estimates $\hat{\beta}_n$ that are the solution of

$$U_n(\beta) = \sum_{i=1}^{n} \omega_{F_n}(X_i) A(\beta; Z_i) \{\hat{\theta}^{\text{IJ}}_{n,i} - \mu(\beta; Z_i)\} = 0.$$

The functions ρ , $\delta^*_{X_i}$ and ω are chosen so that the condition (10) in Theorem 2 is satisfied. In the application to a cohort with left-truncation, the probability measure in the expectations in (10) and (11) corresponds to the measure conditional on truncation.

Theorem 2. *Consider the setup of Proposition 1. Assume $A(\beta; Z_i)$ has finite second moment and the weight functional $f \to \omega_f(\cdot)$ is differentiable with locally Lipschitz continuous derivative, e.g. two times continuous differentiable. Assume further that ω satisfies*

$$E\big[\omega_F(X_i) A(\beta_0; Z_i) \{\phi(\rho(F)) + \phi'_{\rho(F)}(\delta^*_{X_i} - \rho(F)) - \mu(\beta_0; Z_i)\}\big] = 0. \tag{10}$$

Then

$$\frac{1}{\sqrt{n}} U_n(\beta_0) \xrightarrow{d} N(0, \Sigma)$$

where

$$\Sigma = 2^2 \cdot E\{h(\bar{X}_1, \bar{X}_2) h(\bar{X}_1, \bar{X}_3)^T\}, \tag{11}$$

and

$$h(\bar{X}_i, \bar{X}_j) = \frac{1}{2}(h^*_{ij} + h^*_{ji})$$

and

$$\begin{aligned}
h^*_{ij} &= h^*(\bar{X}_i, \bar{X}_j) \\
&= \omega_F(X_i) A(\beta_0; Z_i) \{\phi(\rho(F)) + \phi'_{\rho(F)}(\delta^*_{X_i} - \rho(F)) - \mu(\beta_0; Z_i)\} \qquad (12) \\
&\quad + \omega'_F(X_i; \delta_{X_j} - F) A(\beta_0; Z_i) \{\phi(\rho(F)) + \phi'_{\rho(F)}(\delta^*_{X_i} - \rho(F)) - \mu(\beta_0; Z_i)\} \\
&\quad + \omega_F(X_i) A(\beta_0; Z_i) \phi''_{\rho(F)}(\delta^*_{X_i} - \rho(F), \rho'_F(\delta_{X_j} - F)).
\end{aligned}$$

Proof. Using that weight function $f \to \omega_f$ is differentiable with locally Lipschitz derivative, we may express the estimating function as

$$\begin{aligned}
\frac{1}{n}U_n(\beta_0) &= \frac{1}{n}\sum_{i=1}^{n}\omega_F(X_i)A(\beta_0;Z_i)\{\phi(\rho(F)) + \phi'_{\rho(F)}(\delta^*_{X_i} - \rho(F)) - \mu(\beta_0;Z_i)\} \\
&\quad + \omega'_F(X_i;F_n - F)A(\beta_0;Z_i)\{\phi(\rho(F)) + \phi'_{\rho(F)}(\delta^*_{X_i} - \rho(F)) - \mu(\beta_0;Z_i)\} \\
&\quad + \omega_F(X_i)A(\beta_0;Z_i)\{\phi''_{\rho(F)}(\delta^*_{X_i} - \rho(F), \rho'_F(F_n - F))\} + o_{\mathrm{P}}(n^{-1/2}) \\
&= \frac{1}{n^2}\sum_{i=1}^{n}\sum_{j=1}^{n}\omega_F(X_i)A(\beta_0;Z_i)\{\phi(\rho(F)) + \phi'_{\rho(F)}(\delta^*_{X_i} - \rho(F)) - \mu(\beta_0;Z_i)\} \\
&\quad + \omega'_F(X_i;\delta_{X_j} - F)A(\beta_0;Z_i)\{\phi(\rho(F)) + \phi'_{\rho(F)}(\delta^*_{X_i} - \rho(F)) - \mu(\beta_0;Z_i)\} \\
&\quad + \omega_F(X_i)A(\beta_0;Z_i)\phi''_{\rho(F)}(\delta^*_{X_i} - \rho(F), \rho'_F(\delta_{X_j} - F)) + o_{\mathrm{P}}(n^{-1/2}) \\
&= \frac{1}{n^2}\sum_{i=1}^{n}\sum_{j=1}^{n}h^*_{ij} + o_{\mathrm{P}}(n^{-1/2}) \\
&= \binom{n}{2}^{-1}\sum_{i=1}^{n}\sum_{j<i}\frac{1}{2}(h_{ij} + h_{ji}) + o_{\mathrm{P}}(n^{-1/2}).
\end{aligned} \tag{13}$$

The first term of (13) is a symmetric mean zero U-statistics of order 2. The mean of the first term of (12) is zero by assumption (10). The mean of the second term of (12) is

$$\begin{aligned}
&\mathrm{E}\left(\omega'_F(X_i;\delta_{X_j} - F)A(\beta_0;Z_i)\{\phi(\rho(F)) + \phi'_{\rho(F)}(\delta^*_{X_i} - \rho(F)) - \mu(\beta_0;Z_i)\}\right) \\
&= \mathrm{E}_{\bar{X}_i}\left(\mathrm{E}\left(\omega'_F(x;\delta_{X_j} - F)\right)\big|_{x=X_i}A(\beta_0;Z_i)\{\phi(\rho(F)) + \phi'_{\rho(F)}(\delta^*_{X_i} - \rho(F)) - \mu(\beta_0;Z_i)\}\right) \\
&= 0,
\end{aligned}$$

due to the property of the influence function in (4). The mean of the third term of (12) is

$$\begin{aligned}
&\mathrm{E}\left(\omega_F(X_i)A(\beta_0;Z_i)\phi''_{\rho(F)}(\delta^*_{X_i} - \rho(F), \rho'_F(\delta_{X_j} - F))\}\right) \\
&= \mathrm{E}_{\bar{X}_i}\left(\omega_F(X_i)A(\beta_0;Z_i)\mathrm{E}\left(\phi''_{\rho(F)}(\delta^*_x - \rho(F), \rho'_F(\delta_{X_j} - F))\right)\big|_{x=X_i}\right) \\
&= 0,
\end{aligned}$$

due again to the property of the influence function.

It follows from Theorem 12.3 of van der Vaart (1998) that $n^{-1/2}U_n(\beta_0)$ converges in distribution to a mean zero normal distribution with variance (11). □

The asymptotic distribution of $\hat{\beta}_n$ now follows for standard asymptotic arguments (Parner et al., 2020). We state the result, but leave the proof to the reader.

Theorem 3. *Assume the following regularity conditions*

1. $\mu(\cdot;z)$ *and* $A(\cdot;z)$ *are continuously differentiable for all* $z \in \mathcal{Z}$.
2. $A(\beta_0;Z)$ *has finite second moment.*
3. $\frac{\partial}{\partial\beta}A(\beta;Z)\mu(\beta;Z)$ *and* $A(\beta;Z)\frac{\partial}{\partial\beta}\mu(\beta;Z)$ *are dominated integrated in a neighborhood of* β_0.
4. $|\frac{\partial}{\partial\beta}A(\beta;Z)|$ *is dominated integrable in a neighborhood of* β_0.
5. *The matrix*

$$M = \mathrm{E}\left\{\omega_F(X)A(\beta_0;Z)\frac{\partial}{\partial\beta}\mu(\beta;Z)\big|_{\beta=\beta_0}\right\}$$

is invertible.

Then an estimator $\hat{\beta}_n$ *exists so that* $U_n(\hat{\beta}_n) = 0$ *with a probability tending to* 1 *for* $n \to \infty$. *Moreover,*

$$\sqrt{n}(\hat{\beta}_n - \beta_0) \xrightarrow{d} N\{0, M^{-1}\Sigma(M^{-1})^T\}$$

as $n \to \infty$.

References

Dudley RM, Norvaiša R (1999) Differentiability of six operators on nonsmooth functions and p-variation. Lecture Notes in Mathematics 1703. Springer-Verlag, Berlin. With the collaboration of Jinghua Qian.

Dudley RM, Norvaiša R (2011) Concrete functional calculus. Springer

Johansen S, Gill R (1990) A survey of product-integration with a view towards applications in survival analysis. Annals of Statistics 18:1501–1555

Keller HH (2006) Differential calculus in locally convex spaces. Lecture Notes in Mathematics, vol 417. Springer-Verlag, Berlin-New York

Overgaard M, Parner ET, Pedersen J (2017) Asymptotic theory of generalized estimating equation based on jack-knife pseudo-observations. Annals of Statistics 45(5):1988–2015

Parner ET, Andersen PK, Overgaard M (2020) Cumulative risk regression in case–cohort studies using pseudo-observations. Lifetime Data Analysis pp 1–20

van der Vaart AW (1998) Asymptotic statistics. Cambridge Series in Statistical and Probabilistic Mathematics, Cambridge University Press
